# Confidence in mathematics is confounded by responses to reverse-coded items

**DOI:** 10.3389/fpsyg.2024.1489054

**Published:** 2024-10-24

**Authors:** Faye Antoniou, Mohammed H. Alghamdi

**Affiliations:** ^1^Department of Educational Studies, National and Kapodistrian University of Athens, Athens, Greece; ^2^Department of Self-Development Skills, King Saud University, Riyadh, Saudi Arabia

**Keywords:** reverse coded items, aberrant responding, carelessness, random responding, person-fit indices, lz*, Guttman errors, U3

## Abstract

**Introduction:**

This study investigates the confounding effects of reverse-coded items on the measurement of confidence in mathematics using data from the 2019 Trends in International Mathematics and Science Study (TIMSS).

**Methods:**

The sample came from the Saudi Arabian cohort of 8th graders in 2019 involving 4,515 students. Through mixture modeling, two subgroups responding in similar ways to reverse-coded items were identified representing approximately 9% of the sample.

**Results:**

Their response to positively valenced and negatively valenced items showed inconsistency and the observed unexpected response patterns were further verified using Lz*, U3, and the number of Guttman errors person fit indicators. Psychometric analyses on the full sample and the truncated sample after deleting the aberrant responders indicated significant improvements in both internal consistency reliability and factorial validity.

**Discussion:**

It was concluded that reverse-coded items contribute to systematic measurement error that is associated with distorted item level parameters that compromised the scale’s reliability and validity. The study underscores the need for reconsideration of reverse-coded items in survey design, particularly in contexts involving younger populations and low-achieving students.

## 1 What are the effects of reverse-coded items?

Several authors have pointed to the detrimental effects of reverse-coded items on the quality of the collected data (Roszkowski and Soven, 2010; Weems and Onwuegbuzie, 2001). For example, Clauss and Bardeen (2020) reported that the presence of negatively worded items confounded the conclusions on the simple structure of the Attentional Control Scale (ACS), which, although should be unidimensional it was found to have a bifactor structure (see also Pedersen et al., 2024; Ponce et al., 2022). They concluded that reversing the negatively worded items resulted in producing a method factor rather than an attentional control factor, thus, complicating the conceptual factor structure, and producing an incoherent structure with unexplained item relations (Merritt, 2012; Pedersen et al., 2024). Concerns about internal consistency reliability and content differentiation have also been raised (Roszkowski and Soven, 2010; Weems and Onwuegbuzie, 2001). For example, Jaensson and Nilsson (2017) reported that reversing negatively worded to positively worded items with the same content resulted in a poor to moderate agreement between the two, as was evident from intraclass correlation coefficients between 0.35 and 0.76. Thus, the obtained responses to the same content, using a different item format, resulted in discrepant responses, raising concerns about item scoring and item interpretation. Bolt et al. (2020) reported that reverse-coded items may have deleterious effects on item comprehension. They reported that rates of confusion and misunderstanding of negatively worded items were as high as 53% for students in grades 3 through 5, representing a salient concern for these students as issues of identification, placement, and early intervention are most important at an early age. They further stated that skills and competencies were significantly underestimated in the presence of opposite format items. Similar distortions have been found with adolescents and adults such as teacher and student populations (Barnette, 1996), although non-significant differences across age groups have also been reported (Steinmann et al., 2024).

Interpretations as to the “why’s” of differential responses to the same content as a function of item format have been traced to Personality traits such as agreeableness (Roszkowski and Soven, 2010) or neuroticism (Koutsogiorgi and Michaelides, 2022), the lack of motivation (Kam, 2018), the presence of response styles such as acquiescence (DiStefano and Motl, 2009), situational factors such as carelessness and inattention (Baumgartner et al., 2018; Steinmann et al., 2022; Swain et al., 2008), differential interpretation of item content (Weems and Onwuegbuzie, 2001), low achievement (Steinmann et al., 2024; Weems et al., 2003), mood (Koutsogiorgi and Michaelides, 2022), and demographic variables such as gender with boys having higher levels of inconsistent responses (Marsh and Grayson, 1995; Steedle et al., 2019; Steinmann et al., 2024). However, potential causes are beyond the scope of the present study and will not be discussed in more detail.

### 1.1 What are current recommendations for dealing with reverse-coded items?

At the analytical level, recommendations to deal with reverse-coded items include fitting multidimensional models such as the bifactor model to account for method variance likely attributed to the item format for negatively worded items. Identifying and deleting individuals who behave in unexpected ways as they provide invalid estimates for themselves and harm the psychometric qualities of the measured instrument (Michaelides, 2019). Identifying sources of confusion for reverse-coded items and treating those items through refinement and revision to isolate the sources of confusion has also been recommended, especially for younger age groups (Bolt et al., 2020; Fukudome and Takeda, 2024; Steinmann et al., 2024).

### 1.2 Context and goals of the present study

We selected the examination of confidence ratings for students in Saudi Arabia using the Trends of International Mathematics Assessment (Mullis et al., 2020) international study for several reasons. First, Saudi students are classified among the lowest in mathematics achievement across TIMSS’s participating countries, thus, examination of whether their assessments involve deficits in the psychological sphere of confidence is important. Second, recent data from PISA 2022 indicated that Saudi students had the highest ratings of “straightlining” responses in the survey instruments such as assertiveness, cooperation, etc. Thus, examination of aberrant responses in this population is important, because already approximately 4.5 to 5% of the participant responses are currently screened for and deleted from international databases due to straightlining. Thus, the goals of the present study were (a) to identify subgroups of participants who respond in the same manner in reverse-coded items using mixture modeling, (b) to validate the identification of aberrance mixtures using person fit indicators, (c) to test the presence of systematic error variance through supporting an additional “method” factor, and (d) evaluate the effects of aberrant responders on the psychometrics of a confidence scale related to mathematics achievement contrasting original and purified data (after deleting aberrant responders).

## 2 Methods

### 2.1 Participants and procedures

Participants were 5,680 Saudi 8^th^-grade students who took part in the 2019 TIMSS study. There were 2,884 males (50.8%) and 2,791 females (49.2%). Most students (91.7%) attended public schools with a small percentage (8.3%) attending international schools. The mean age was 13.926 years (SD = 0.679). In the TIMSS 2019 study, students in each country are selected for participation using multistage stratified random sampling to achieve representativeness to the population and specifically the characteristics of national student populations regarding geographic regions and school types. Sample sizes are more than 4,000 students and sampling engages at least 150 schools. The TIMSS guidelines also state clear guidelines to ensure high participation during the testing process, and to avoid biased samples that threaten generalization of the findings to the population. More details on the study and its methodology can be traced.^1^^,^^2^

### 2.2 Measure

**Figure 1:**
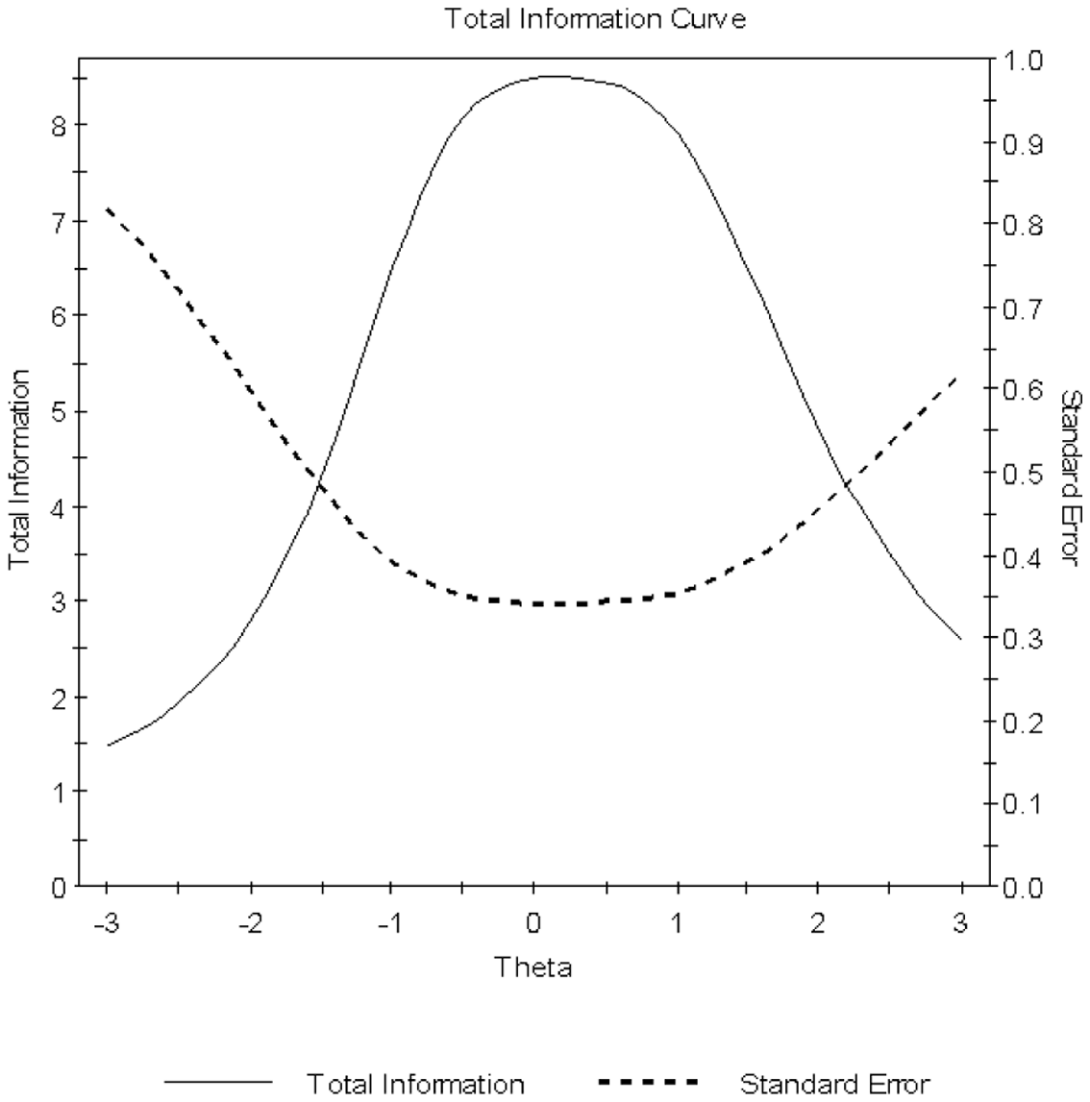
Total information curve and conditional standard error of measurement for the mathematics confidence scale.

The “Students Confidence in Mathematics scale” was implemented which comprises nine items. Example items that are positively worded were “I usually do well in mathematics,” “I learn things quickly in mathematics,” and “I am good at working out difficult mathematics problems.” Sample negatively worded items were “Mathematics makes me nervous” and “Mathematics makes me confused. Respondents indicated their agreement with each statement on a four-point Likert scale ranging from “Agree a lot” to “Disagree a lot” with no midpoint option. Higher scores on the confidence scale indicate greater confidence in mathematics. Internal consistency reliability was assessed using Cronbach’s alpha coefficient and was 0.81. Based on TIMSS 2019, the scale is unidimensional, and scale scores are provided per country along with cutoff scores. Our country-based analysis using the Graded Response Model (GRM) showed marginal reliability estimates equal to 0.86 and adequate omnibus model fit via the Root Mean Squared Error of Approximation (RMSEA) that was equal to 0.08, after reversing the items that have the opposite meaning to reflect positive covariances across all items and post purification (i.e., after deleting aberrant responders). Figure 1 displays the Test Information Function (TIF) and corresponding Conditional Standard Error of Measurement (CSEM) of the scale which shows a nice coverage of information across ±2.5 theta scores and a center around the mean of zero as expected. Further analyses of category information curves are shown in the Appendix which support the used scaling system with no overlap or disordering.

### 2.3 Data analyses

#### 2.3.1 Criteria for classifying participants to groups

The following three criteria were utilized to evaluate model fit in the classification process. Lower values are indicative of better model fit. Although entropy refers to the estimation of the indices below, in a standalone form, entropy is not included in the process of concluding the most optimal latent class as earlier suggested (Masyn, 2013).

The Classification Likelihood Criterion (CLC) is a measure of model fit that considers both the log-likelihood of the model and the entropy of the classification. Entropy reflects the uncertainty of the in-class assignments, with higher entropy indicating more uncertainty (i.e., lower classification certainty). The goal of CLC is to find a balance between model fit (log-likelihood) and classification certainty (entropy). CLC is calculated as shown in Equation (1) below:


(1) $$CLC=-2^{*}\left(LL-\text{Entropy}\right)$$

With LL being the log-likelihood of the model, and Entropy reflecting uncertainty in the classification of participants to subgroups.

The Akaike Weight of Evidence (AWE) is a model selection criterion that combines information about model fit (log-likelihood) and penalizes model complexity more strongly than the traditional Akaike Information Criterion (AIC). It favors simpler models unless the additional layers of complexity are justified based on omnibus model fit indicators. It is estimated as shown in Equation (2) below:


(2) $$\text{AWE}=-2^{*}LL+2^{*}m^{*}\log\left(\log\left(n\right)\right)+2^{*}\text{Entropy}$$

With LL being the log-likelihood of the model, *m* is the number of estimated parameters, and *n is* the sample size.

The Integrated Classification Likelihood-Bayesian Information Criterion (ICL-BIC) is a variant of the Bayesian Information Criterion (BIC) that also accounts for the quality of classification. Thus, the ICL-BIC criterion aims to select models that not only have a good fit (as indicated by the BIC) but also provide clear and distinct class memberships (as indicated by entropy). It is defined as shown in Equation (3) below:


(3) ICL−BIC=BIC−Entropy

With BIC being the Bayesian Information Criterion, calculated as BIC = −2*LL + m*log(*n*), and Entropy the classification uncertainty.

#### 2.3.2 Validating person aberrant behavior via analyzing response patterns

Three of the most prominent person fit indicators namely, U3, Guttman errors, and lz* were selected to validate the results from the latent class analyses (Emons, 2008; Cui and Mousavi, 2015). Each of these indices has its advantages and limitations or is sensitive to specific patterns of aberrance. The U3 statistic provides a nuanced assessment of aberrant responding, being sensitive to subtle deviations from the Guttman pattern (Schroeders et al., 2021). It is most efficacious in detecting inattention. Its disadvantage, however, is its sensitivity to test length, with brief measures jeopardizing its reliability. The number of Guttman errors offers a traditional measure of misfit by examining the type of response as a function of item difficulty (Meijer, 1994; Tendeiro and Meijer, 2014). It is easy to understand but it may not be sensitive to more subtle forms of misfit. The lz* statistic is a powerful tool for detecting misfits in response patterns and contributes to a comprehensive analysis of response data. The lz* statistic has been found effective in detecting various types of aberrant responding, such as fake good and random responding (Avşar, 2022; Beck et al., 2019; Karabatsos, 2003). In terms of their direction, low values in lz* (i.e., <−1.3) are indicative of aberrance and the opposite is true for the number of Guttman errors (G) and U3 for which larger values are indicative of aberrant responding. All person fit analyses were conducted using the Perfit package (Tendeiro et al., 2016) in R (Team R. C, 2015).

#### 2.3.3 Ancillary analyses involving confirmatory factor analyses (or the graded response model)

Several CFA models or the Graded Response Model (GRM) were fit to the data to estimate unidimensionality, the presence of a methods factor, item-level statistics, and omnibus model fit. A preferred index in all these tests was the Root Mean Squared Error of Approximation (RMSEA) for which values less than 0.08 signal acceptable model fit. In CFA descriptive fit indices such as the Comparative Fit Index (CFI) need to take on values greater than 0.900. To ensure that the sample size was adequate we conducted a Monte Carlo simulation positing a unidimensional construct with 9 items, factor loadings equal to 0.70 and residual variances equal to 0.51. Using either the full or truncated samples parameter recovery ranged between 95 and 96%, and power for the factor loadings was greater than 99.9%. The chi-square statistic was overpowered, which is why it was not relied upon when evaluating model fit.

## 3 Results

### 3.1 Identification of aberrance using mixture modeling

**Figure 2:**
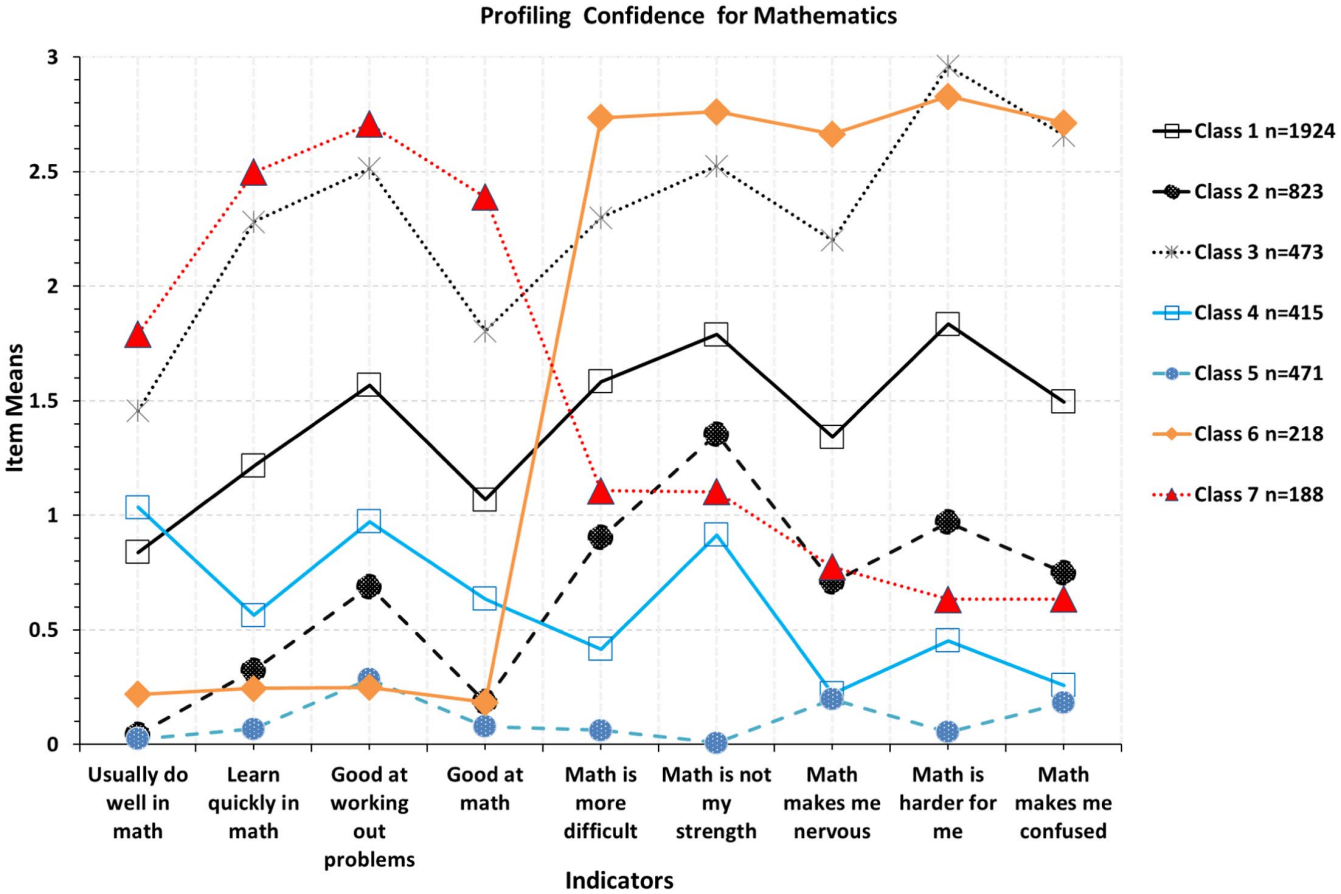
Optimal latent class solution for the measurement of confidence for mathematics in Saudi Arabia.

Figure 2 displays an optimal solution judged by the above fit indices and a minimum sample size of *n* > 50 participants, selected so that sample representation would be greater than 1%. This latter criterion is justified because subgroups with n < =50 may not represent true subgroups in the population but rather artifacts of the sampling process. Based on the above criteria, a 7-class solution was superior to an 8-class solution as indices of AWE and ICL-BIC were smaller for the 7-class model compared to the 8-class model but not so for the CLC (AWE_7-class_ = 96866.600; AWE_8-class_ = 97023.894; ICL-BIC_7-class_ = 95873.538; ICL-BIC_8-class_ = 95916.688; CLC_7-class_ = 95141.478; CLC_8-class_ = 95100.482). Similarly, the 7-class model had lower values compared to the 6-class model supporting its preference for the CLC and ICL-BIC only (AWE_7-class_ = 96866.600; AWE_6-class_ = 96848.480; ICL-BIC_7-class_ = 95873.538; ICL-BIC_6-class_ = 95969.564; CLC_7-class_ = 95141.478; CLC_6-class_ = 95321.648). As shown in the figure, classes 6 and 7 represent aberrant responders reflecting low and high confidence ratings, respectively. Given that the scores were reversed for the LCA analysis, the expectation is that the same direction/scoring of the items would reflect ratings that are similar across items, expecting horizontal lines that are parallel to the X-axis. Differences from a “flat” line would be indicative of content differences regarding confidence and should be expected. However, as seen in class 6, mean responses to the first four items (about being confident in math) were very low followed by very high levels in the items describing lack of confidence. However, as mentioned above, given that items were reverse coded for the analysis, the direction for all items was the same, and thus, the difference in ratings of that magnitude is likely indicative of inattention, carelessness, random responding, or other personal or situational factors reflecting some form of systematic error of measurement. In other words, participants provided the same rating (e.g., agreement) for items such as “I am good at math” and “Mathematics is not my strength,” which shows inconsistency and error. The same was true for latent class 7, for which confidence ratings were high in math followed by very low ratings which again, is incongruent given the same direction of items in this presentation (items were reverse-coded).

### 3.2 Validating aberrance using person-fit indicators

**Figure 3:**
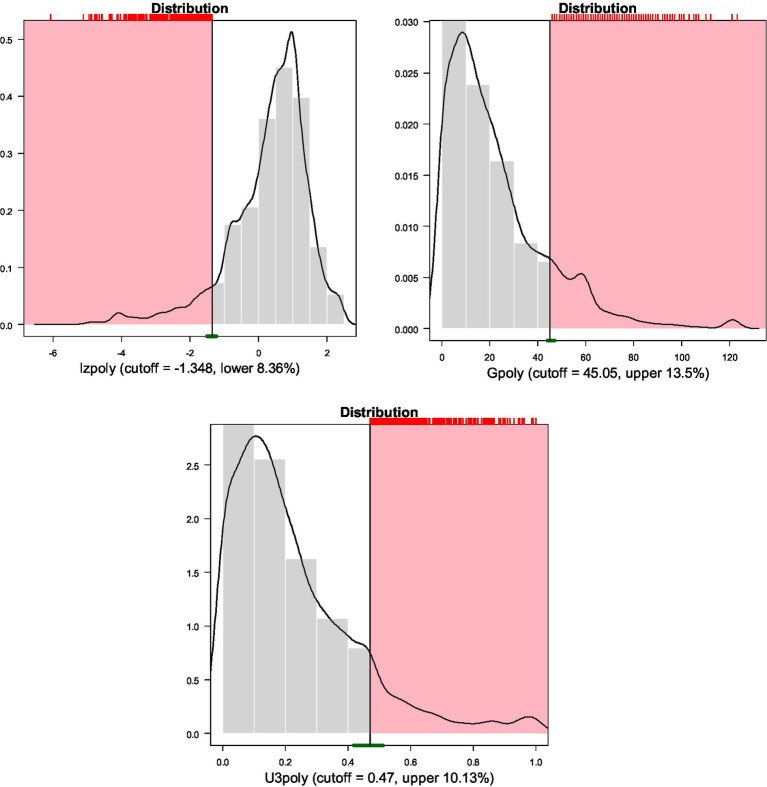
Cutoff values for indices of aberrant responding.

**Figure 4:**
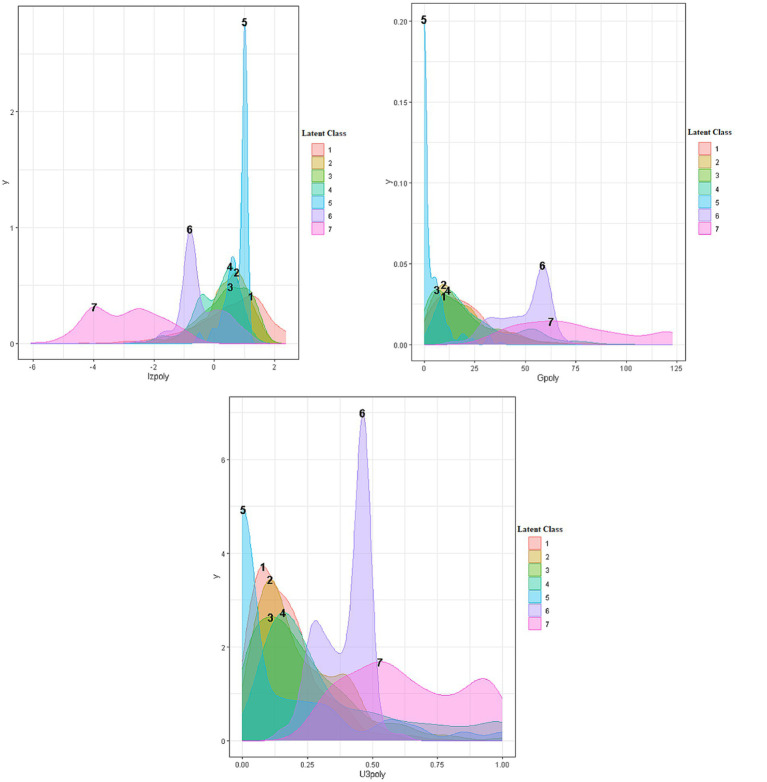
Densities of person-fit indices per latent class in the 7-class optimal solution (modal).

Figure 3 displays densities and cutoff values using a cutoff threshold of 10% (or 90% depending on whether low or high scores were considered aberrant) for the three person-fit indices. As shown in the figure, the cutoff value for the lz* indicator was −1.348, for the number of Guttman errors 45.05, and for the U3 indicator 0.47. Figure 4 provides densities for the three person-fit indicators by latent class. A means analysis using the Analysis of Variance (ANOVA) model was run to identify and contrast point estimates across the 7 classes. Results pointed to significant differences between groups using the omnibus F-test for Lz* [*F*(6, 4,510) = 456.926, *p* < 0.001], Guttman errors [*F*(6, 4,511) = 594.943, *p* < 0.001] and the U3 index [*F*(6, 4,511) = 273.023, *p* < 0.001]. Using Tukey’s *post-hoc* tests results indicated that the two aberrant classes (i.e., 6 and 7) were significantly more aberrant compared to all other classes. When contrasted with each other, class 7 estimates of aberrance were significantly elevated compared to class 6 estimates. Using the eta-squared effect size metric, differences between classes 6 and 7 and all other classes ranged between 0.27 and 0.44, reflecting larger-than-large effects (large eta squared =0.14; Cohen, 1988; Lakens, 2013).

When evaluating differences using the Figure 3 cutoff values it is evident that the mean number of Guttman errors for classes 6 and 7 that were 74 and 49, respectively, exceed the critical values of 45 suggesting that most participants were aberrant responders emitting a significant number of Guttman errors. Regarding the lz* and U3 indices, class 7 had mean estimates (MeanLz* = 2.925; MeanU3 = 0.633) much greater than the thresholds defining aberrant responding. For class 6, mean point estimates were close to the cutoff values but lower (MeanLz* = −0.449; MeanU3 = 0.395).

### 3.3 Was the observed aberrance a function of a methods factor?

This hypothesis was tested by contrasting a unidimensional versus a two-factor model with the latter separating positively worded from negatively worded items. After fitting the data to a unidimensional measurement model using the Weighted Least Squares Mean and Variance adjusted (WLSMV) estimator that is appropriate for ordered data, results indicated that all items loaded significantly to their respective factor (at *p* < 0.001) but the global model fit was poor (CFI = 0.824, RMSEA = 0.210). On the contrary, separating the items based on valence into positively worded and negatively worded factors resulted in improved and acceptable model fit (CFI = 0.974; RMSEA = 0.078). Interestingly, the correlation between factors was *r* = 0.492 using Pearson’s r. These findings point to the existence of a methods factor that accounted for the intercorrelation between items due to item wording.

### 3.4 Evaluating scale psychometrics using original and purified data

Using a purification procedure, analyses of internal consistency reliability and factorial validity were conducted contrasting the results from the full sample against those from a truncated sample that excluded the participants from classes 6 and 7. Regarding internal consistency reliability results indicated that the full sample estimate was 0.820 and was elevated to 0.837 for the truncated sample. When contrasting the two coefficients using a Fisher’s z transformation Z-test (Hinkle et al., 1988), results pointed to significant improvements in internal consistency reliability for the truncated sample (*Z* = 2.542, *p* = 0.011).

Using the Confirmatory Factor Analysis (CFA) model results indicated that model fit was improved using the truncated data compared to the full sample (e.g., CFI_Full_ = 0.824; CFI_Truncated_ = 0.890). Interestingly, when contrasting models using the RMSEA, the confidence intervals for the RMSEA using full data at 95% ranged between 0.205 and 0.214. The point estimate for the RMSEA using truncated data was 0.167 and its respective 95% confidence interval ranged between 0.162 and 0.171. Thus, the point estimate of the RMSEA using the truncated data was significantly different from the one using full data as the confidence interval for the full data did not include the point estimate of the RMSEA using truncated data.

## 4 Discussion

The goals of the present study were (a) to identify subgroups of participants who respond in the same manner in reverse-coded items using mixture modeling, (b) to validate the identification of aberrance mixtures using person fit indicators, (c) to test the presence of systematic error variance through supporting an additional “method” factor, and (d) evaluate the effects of aberrant responders on the psychometrics of a confidence scale related to mathematics achievement.

One important finding was that two classes of individuals who responded in the same manner across reverse-coded items were identified using mixture modeling, one with low confidence and one with high confidence for mathematics. Furthermore, an analysis of these two groups using person fit indicators showed that mean levels of aberrance were significantly elevated in these two groups, compared to the remaining five subgroups. This finding provides further support for using mixture modeling to identify subgroups that potentially behave in aberrant ways. The concordance between person-fit indicators and the subgrouping produced via the LCA analysis was high, validating the use of person-based analyses. Furthermore, the combination of participants in classes 6 and 7 represented 9% of the sample. This magnitude is lower compared to unpublished data from Bandalos, Coleman, and Gerstner (cited in Reise et al., 2016) who reported rates of inconsistent responses to positively worded and negatively worded items in the Rosenberg Self-Esteem scale (RSES) at rates between 12 and 17%.

Another important finding was that the inclusion of these two subgroups had important negative implications for the scale’s psychometric analyses. An inferential statistical test indicated significant improvements in internal consistency reliability using the truncated sample compared to the full sample. Similarly, by employing the 95% confidence intervals of the RMSEA significant differences in model fit were present, with better model fit being associated with the truncated dataset. This finding agrees with earlier work in that negatively worded items were associated with poor psychometric characteristics such as enhanced item difficulty levels and lower discriminant ability compared to positively worded items, as per the IRT model (Kam, 2023; Sliter and Zickar, 2014). Thus, the idea that by reversing negatively worded items they function in equivalent ways with their positive counterparts simply does not hold.

A third finding was that the integration of person-based, and variable-based analysis contributed to our conclusion that positively worded and negatively worded items represent distinct facets due to item wording, representing method variance rather than distinct facets of the underlying confidence construct. In Marsh et al. (2010) terms this systematic form of variance represents “ephemeral” variance. Using the factor model, results indicated that a 2-factor solution favored the unidimensional model with all positively and all negatively worded items loading on two distinct dimensions. This finding agrees with past studies that a method effects factor was identified (e.g., Kam et al., 2021; Podsakoff et al., 2003).

### 4.1 Recommendations limitations and future directions

Based on the empirical evidence described above and the current empirical findings it is suggested that reverse-coded items should be avoided as they may be associated with erroneous responses, carelessness, lack of understanding, or the tendency to satisfice (Krosnick, 1991; Suárez-Álvarez et al., 2018). The empirical evidence has suggested that their inclusion likely contributes to spurious rather than substantive measurements (factors) due to item format (positive versus negative phrasing; Barnette, 1996). Thus, the results from the present study add to previous recommendations that the practice of including negatively worded items in surveys and self-report instruments may introduce artificial methods effects and needs to be avoided as it may compromise both content and construct validity (Marsh, 1996; Domínguez-Salas et al., 2022). Specifically, effects of negatively worded items on cognitive fatigue or lack of cognitive reflection have been documented (Fukudome and Takeda, 2024; Koutsogiorgi and Michaelides, 2022; Merritt, 2012) or have been linked to specific personality types (Weems et al., 2003; DiStefano and Motl, 2009) such as the behavioral inhibition system (BIS, Quilty et al., 2006) or behavioral activation systems (BAS, Weydmann et al., 2020). On the opposite side of this argument, however, Vigil-Colet et al. (2020) suggested that reversed coded items could be valuable and could be used in survey measurement only if acquiescence and response biases are controlled for statistically. A series of novel methodologies are currently available in that regard (Plieninger and Heck, 2018; Machado et al., 2024).

The present study is limited for several reasons. First, there were no direct and observable indicators of aberrant responding; instead, aberrance was inferred from the person fit indices as they reflect deviations between observed and expected responses to items based on adherence to the Guttman pattern. The use of additional measurements such as eye-tracking, cognitive, or self-report measures could provide additional insight into the causes behind inconsistent responses in negatively valenced items.

In the future, it will be important to devise methodologies to both identify and correct estimates for the presence of aberrant responses due to reverse-coded items. Bolt et al. (2020) presented an IRT mixture model that makes use of Samejima’s (1969) Graded Response Model (GRM) to identify what they termed as “confused” classes. They presented models to identify full confusion, utilizing all items of a scale, or partial confusion utilizing half of the items. Kam (2018) proposed the latent difference (LD) modeling approach from Pohl et al. (2008) to identify method effects. Further combinations of mixture models that model separate ability and aberrance and adjust person-ability estimates may also be useful (Yamamoto, 1989). Garcia-Pardina et al. (2024) proposed a new model to estimate “substantive dimensionality” which accommodated variance due to wording effects which entailed exploratory graph analysis (EGA) and parallel analysis (PA). Kam and Fan (2020) proposed multitrait-multimethod methodologies within the factor mixture models to capture heterogeneous responses and Kam and Meyer (2022) suggested applying non-linear methodologies. Last, the inclusion of advanced technologies may also provide additional evidence on explanatory factors (Koutsogiorgi and Michaelides, 2022).

## Data Availability

Publicly available datasets were analyzed in this study. This data can be found at: https://timssandpirls.bc.edu/timss2019/.

## Footnotes

^1^ https://www.iea.nl/studies/iea/timss/2019

^2^ https://timssandpirls.bc.edu/timss2019/

## Appendix

Category information curves as per the GRM model fitted to the confidence data.

## References

[ref1] Avşar A. Ş. (2022). Aberrant individuals’ effects on fit indices both of confirmatory factor analysis and polytomous IRT models. Curr Psychol 41, 7427–7440. doi: 10.1007/s12144-021-01563-4

[ref2] Barnette J. J. (1996). Responses that may indicate nonattending behaviors in three self-administered educational surveys. Res Sch 3, 49–59.

[ref3] Baumgartner H. Weijters B. Pieters R. (2018). Misresponse to survey questions: a conceptual framework and empirical test of the effects of reversals, negations, and polar opposite core concepts. J Mark Res 55, 869–883. doi: 10.1177/0022243718811848

[ref4] Beck M. F. Albano A. D. Smith W. M. (2019). Person-fit as an index of inattentive responding: a comparison of methods using polytomous survey data. Appl Psychol Meas 43, 374–387. doi: 10.1177/0146621618798666 31235983 PMC6572906

[ref5] Bolt D. Wang Y. C. Meyer R. H. Pier L. (2020). An IRT mixture model for rating scale confusion associated with negatively worded items in measures of social-emotional learning. Appl Meas Educ 33, 331–348. doi: 10.1080/08957347.2020.1789140

[ref6] Clauss K. Bardeen J. R. (2020). Addressing psychometric limitations of the attentional control scale via bifactor modeling and item modification. J Pers Assess 102, 415–427. doi: 10.1080/00223891.2018.1521417, PMID: 30398371

[ref7] Cohen J. (1988). Statistical power analysis for the behavioral sciences. 2nd Edn. New York: Lawrence Erlbaum Associates.

[ref8] Cui Y. Mousavi A. (2015). Explore the usefulness of person-1t analysis on large-scale assessment. Int J Test 15, 23–49. doi: 10.1080/15305058.2014.977444

[ref9] DiStefano C. Motl R. W. (2009). Personality correlates of method effects due to negatively worded items on the Rosenberg self-esteem scale. Personal Individ Differ 46, 309–313. doi: 10.1016/j.paid.2008.10.020, PMID: 37217890

[ref10] Domínguez-Salas S. Andrés-Villas M. Riera-Sampol A. Tauler P. Bennasar-Veny M. Aguilo A.. (2022). Analysis of the psychometric properties of the sense of coherence scale (SOC-13) in patients with cardiovascular risk factors: a study of the method effects associated with negatively worded items. Health Qual Life Outcomes 20, 1–14. doi: 10.1186/s12955-021-01914-6 35012547 PMC8751372

[ref11] Emons W. (2008). Nonparametric person-fit analysis of polytomous item scores. Appl Psychol Meas 32, 224–247. doi: 10.1177/0146621607302479

[ref12] Fukudome K. Takeda T. (2024). The influence of cognitive reflection on consistency of responses between reversed and direct items. Personal Individ Differ 230:112811. doi: 10.1016/j.paid.2024.112811

[ref13] Garcia-Pardina A. Abad F. J. Christensen A. P. Golino H. Garrido L. E. (2024). Dimensionality assessment in the presence of wording effects: a network psychometric and factorial approach. Behav Res Methods 56, 6179–6197. doi: 10.3758/s13428-024-02348-w 38379114

[ref14] Hinkle D. E. Wiersma W. Jurs S. G. (1988). Applied statistics for the behavioral sciences. 2nd Edn. Boston: Houghton Mifflin Company.

[ref15] Jaensson M. Nilsson U. (2017). Impact of changing positively worded items to negatively worded items in the Swedish web-version of the quality of recovery (SwQoR) questionnaire. J Eval Clin Pract 23, 502–507. doi: 10.1111/jep.12639, PMID: 27650792

[ref16] Kam C. C. S. (2018). Novel insights into item keying/valence effect using latent difference modeling analysis. J Pers Assess 100, 389–397. doi: 10.1080/00223891.2017.1369095, PMID: 28980826

[ref17] Kam C. C. S. (2023). Why do regular and reversed items load on separate factors? Response difficulty vs. item extremity. Educ Psychol Meas 83, 1085–1112. doi: 10.1177/00131644221143972, PMID: 37974659 PMC10638982

[ref18] Kam C. C. S. Fan X. (2020). Investigating response heterogeneity in the context of positively and negatively worded items by using factor mixture modeling. Organ Res Methods 23, 322–341. doi: 10.1177/1094428118790371

[ref19] Kam C. C. S. Meyer J. P. (2022). Testing the nonlinearity assumption underlying the use of reverse-keyed items: a logical response perspective. Assessment 30, 1569–1589. doi: 10.1177/10731911221106775 35818170

[ref20] Kam C. C. S. Meyer J. P. Sun S. (2021). Why do people agree with both regular and reversed items? A logical response perspective Assessment 28, 1110–1124. doi: 10.1177/10731911211001931, PMID: 33779309

[ref21] Karabatsos G. (2003). Comparing the aberrant response detection performance of thirty-six person-fit statistics. Appl Meas Educ 16, 277–298. doi: 10.1207/S15324818AME1604_2

[ref22] Koutsogiorgi C. C. Michaelides M. P. (2022). Response tendencies due to item wording using eye-tracking methodology accounting for individual differences and item characteristics. Behav Res Methods 54, 2252–2270. doi: 10.3758/s13428-021-01719-x 35032021

[ref23] Krosnick J. A. (1991). Response strategies for coping with the cognitive demands of attitude measures in surveys. Appl Cogn Psychol 5, 213–236. doi: 10.1002/acp.2350050305

[ref24] Lakens D. (2013). Calculating and reporting effect sizes to facilitate cumulative science: a practical primer for t-tests and ANOVAs. Front Psychol 4:863. doi: 10.3389/fpsyg.2013.00863, PMID: 24324449 PMC3840331

[ref25] Machado G. M. Hauck-Filho N. Pallini A. C. Dias-Viana J. L. Chiappetta Santana L. H. B. Medeiros da Silva C. A. N.. (2024). Investigating the acquiescent responding impact in empathy measures. Int J Test. 24, 1–26. doi: 10.1080/15305058.2024.2364170

[ref26] Marsh H. W. (1996). Positive and negative self-esteem: a substantively meaningful distinction or artifactors? J Pers Soc Psychol 70, 810–819. doi: 10.1037/0022-3514.70.4.810, PMID: 8636900

[ref27] Marsh H. W. Grayson D. (1995). “Latent variable models of multitrait-multimethod data” in Structural equation modeling: Concept, issues, and applications. ed. Hoyle R. H. (Thousand Oaks, CA: Sage), 177–198.

[ref28] Marsh H. W. Scalas L. F. Nagengast B. (2010). Longitudinal tests of competing factor structures for the Rosenberg self-esteem scale: traits, ephemeral artifacts, and stable response styles. Psychol Assess 22, 366–381. doi: 10.1037/a0019225, PMID: 20528064

[ref9111] Masyn K. E. (2013). Latent class analysis and finite mixture modeling. In: The Oxford handbook of quantitative methods: Statistical analysis. Ed. Little T. D. (Oxford University Press), pp. 551–611.

[ref29] Meijer R. R. (1994). The number of guttman errors as a simple and powerful person-1t statistic. Appl Psychol Meas 18, 311–314. doi: 10.1177/014662169401800402

[ref30] Merritt S. M. (2012). The two-factor solution to Allen and Meyer’s (1990) affective commitment scale: effects of negatively worded items. J Bus Psychol 27, 421–436. doi: 10.1007/s10869-011-9252-3

[ref31] Michaelides M. P. (2019). Negative keying effects in the factor structure of TIMSS 2011 motivation scales and associations with reading achievement. Appl Meas Educ 32, 365–378. doi: 10.1080/08957347.2019.1660349

[ref9001] Mullis I. V. S. Martin M. O. Foy P. Kelly D. L. Fishbein B. (2020). TIMSS 2019 international results in mathematics and science. International Association for the Evaluation of Educational Achievement (IEA). Available at: https://timssandpirls.bc.edu/timss2019/international-results/

[ref32] Pedersen H. S. Christensen K. S. Prior A. Christensen K. B. (2024). The dimensionality of the perceived stress scale: the presence of opposing items is a source of measurement error. J Affect Disord 344, 485–494. doi: 10.1016/j.jad.2023.10.109, PMID: 37852582

[ref33] Plieninger H. Heck D. W. (2018). A new model for acquiescence at the interface of psychometrics and cognitive psychology. Multivar Behav Res 53, 633–654. doi: 10.1080/00273171.2018.1469966, PMID: 29843531

[ref34] Podsakoff P. M. MacKenzie S. B. Lee J. Y. Podsakoff N. P. (2003). Common method biases in behavioral research: a critical review of the literature and recommended remedies. J Appl Psychol 88, 879–903. doi: 10.1037/0021-9010.88.5.879, PMID: 14516251

[ref35] Pohl S. Steyer R. Kraus K. (2008). Modeling method effects as individual causal effects. J R Stat Soc Ser A 171, 41–63. doi: 10.1111/j.1467-985X.2007.00517.x, PMID: 39375710

[ref36] Ponce F. P. Torres Irribarra D. Vergés Á. Arias V. B. (2022). Wording effects in assessment: missing the trees for the forest. Multivar Behav Res 57, 718–734. doi: 10.1080/00273171.2021.1925075, PMID: 34048313

[ref37] Quilty L. C. Oakman J. M. Risko E. (2006). Correlates of the Rosenberg self-esteem scale method effects. Struct Equ Model 13, 99–117. doi: 10.1207/s15328007sem1301_5, PMID: 39256755

[ref38] Reise S. P. Kim D. S. Mansolf M. Widaman K. F. (2016). Is the bifactor model a better model or is it just better at modeling implausible responses? Application of iteratively reweighted least squares to the Rosenberg self-esteem scale. Multivar Behav Res 51, 818–838. doi: 10.1080/00273171.2016.1243461, PMID: 27834509 PMC5312782

[ref39] Roszkowski M. J. Soven M. (2010). Shifting gears: consequences of including two negatively worded items in the middle of a positively worded questionnaire. Assess Eval High Educ 35, 117–134. doi: 10.1080/02602930802618344

[ref9002] Samejima F. (1969). Estimation of latent ability using a response pattern of graded scores. Psychometrika Monograph Supplement, 34:100.

[ref40] Schroeders U. Schmidt C. Gnambs T. (2021). Detecting careless responding in survey data using stochastic gradient boosting. Educ Psychol Meas 82, 29–56. doi: 10.1177/00131644211004708 34992306 PMC8725053

[ref42] Sliter K. A. Zickar M. J. (2014). An IRT examination of the psychometric functioning of negatively worded personality items. Educ Psychol Meas 74, 214–226. doi: 10.1177/0013164413504584

[ref43] Steedle J. T. Hong M. Cheng Y. (2019). The effects of inattentive responding on construct validity evidence when measuring social–emotional learning competencies. Educ Meas Issues Pract 38, 101–111. doi: 10.1111/emip.12256

[ref44] Steinmann I. Chen J. Braeken J. (2024). Who responds inconsistently to mixed-worded scales? Differences by achievement, age group, and gender. Assess Educ Principles, Policy & Practice 31, 5–31. doi: 10.1080/0969594X.2024.2318554

[ref45] Steinmann I. Strietholt R. Braeken J. (2022). A constrained factor mixture analysis model for consistent and inconsistent respondents to mixed-worded scales. Psychol Methods 27, 667–702. doi: 10.1037/met0000392, PMID: 33829811

[ref46] Suárez-Álvarez J. Pedrosa I. Lozano L. M. García-Cueto E. Cuesta M. Muñiz J. (2018). Using reversed items in Likert scales: a questionable practice. Psicothema 30, 149–158. doi: 10.7334/psicothema2018.33, PMID: 29694314

[ref47] Swain S. D. Weathers D. Niedrich R. W. (2008). Assessing three sources of misresponse to reversed Likert items. J Mark Res 45, 116–131. doi: 10.1509/jmkr.45.1.116

[ref48] Team R. C (2015). R: A language and environment for statistical computing. Vienna, Austria: R Foundation for Statistical Computing, Vienna, Austria.

[ref49] Tendeiro J. Meijer R. (2014). Detection of invalid test scores: the usefulness of simple nonparametric statistics. J Educ Meas 51, 239–259. doi: 10.1111/jedm.12046

[ref50] Tendeiro J. N. Meijer R. R. Niessen A. S. M. (2016). PerFit: an R package for person-fit analysis in IRT. J Stat Softw 74, 1–27. doi: 10.18637/jss.v074.i05

[ref51] Vigil-Colet A. Navarro-González D. Morales-Vives F. (2020). To reverse or to not reverse Likert-type items: that is the question. Psicothema 32, 108–114. doi: 10.7334/psicothema2019.286, PMID: 31954423

[ref53] Weems G. H. Onwuegbuzie A. J. (2001). The impact of midpoint responses and reverse coding on survey data. Meas Eval Couns Dev 34:166. doi: 10.1080/07481756.2002.12069033

[ref54] Weems G. H. Onwuegbuzie A. J. Lustig D. (2003). Profiles of respondents who respond inconsistently to positively-and negatively-worded items on rating scales. Evaluation Res Educ 17, 45–60. doi: 10.1080/14664200308668290

[ref55] Weems G. H. Onwuegbuzie A. J. Schreiber J. B. Eggers S. J. (2003). Characteristics of respondents who respond differently to positively and negatively worded items on rating scales. Assess Eval High Educ 28, 587–604. doi: 10.1080/0260293032000130234

[ref56] Weydmann G. Filho N. H. Bizarro L. (2020). Acquiescent responding can distort the factor structure of the BIS/BAS scales. Personal Individ Differ 152:109563. doi: 10.1016/j.paid.2019.109563

[ref57] Yamamoto K. Y. (1989). HYBRID model of IRT and latent class models. Princeton, NJ: Educational Testing Service.

